# Factors Determining Transmission of Persistent Viruses by *Bemisia tabaci* and Emergence of New Virus–Vector Relationships

**DOI:** 10.3390/v13091808

**Published:** 2021-09-11

**Authors:** Saptarshi Ghosh, Murad Ghanim

**Affiliations:** Department of Entomology, Volcani Center, Rishon LeZion 750-5101, Israel; sunnysaptarshi@gmail.com

**Keywords:** circulative transmission, *Bemisia tabaci*, insect vector, virus emergence

## Abstract

Many plant viruses depend on insect vectors for their transmission and dissemination. The whitefly *Bemisia tabaci* (Hemiptera: Aleyrodidae) is one of the most important virus vectors, transmitting more than four hundred virus species, the majority belonging to begomoviruses (*Geminiviridae*), with their ssDNA genomes. Begomoviruses are transmitted by *B. tabaci* in a persistent, circulative manner, during which the virus breaches barriers in the digestive, hemolymph, and salivary systems, and interacts with insect proteins along the transmission pathway. These interactions and the tissue tropism in the vector body determine the efficiency and specificity of the transmission. This review describes the mechanisms involved in circulative begomovirus transmission by *B. tabaci*, focusing on the most studied virus in this regard, namely the tomato yellow leaf curl virus (TYLCV) and its closely related isolates. Additionally, the review aims at drawing attention to the recent knowhow of unorthodox virus—*B. tabaci* interactions. The recent knowledge of whitefly-mediated transmission of two recombinant poleroviruses (*Luteoviridae*), a virus group with an ssRNA genome and known to be strictly transmitted with aphids, is discussed with its broader context in the emergence of new whitefly-driven virus diseases.

## 1. Introduction

Transmission by insects accounts for the spread of over 75% of the known plant-infecting viruses [1]. The mechanisms of transmission and route of plant viruses inside their insect vectors vary depending whether the virus is transmitted in a non-persistent, semi-persistent, persistent non-propagative, or persistent propagative mode [2]. Insect transmissibility is co-regulated by the virus and the insect on a genetic base, with insect–virus protein interactions underlying pathogen transmission [3,4,5]. Persistent transmission of plant viruses involves stringent virus–insect vector interactions, often ensuring that a virus is transmissible by a single insect species. However, this virus–insect specificity, combined with compatibility requirements of the insect vector with the host plant, impedes evolution of new virus–vector relationships [6,7]. For example, transmission of a virus by a new insect is constrained by the required ability of the new vector to feed on the host plant that harbors the evolving virus [8]. Additionally, a successful infection of a virus following its introduction into a new host plant suitable for the new vector is dependent on the ability of the virus to replicate inside the new host plant [8].

The global whitefly (Aleyrodidae) fauna comprises over 160 different genera [9], but only the three genera of *Bemisia* Quaintance and Baker, *Trialeurodes* Cockerell, and *Aleurodicus* Russell are known transmitters of over four hundred diverse viruses (Table 1). *Bemisia tabaci* Gennadius (*B. tabaci*), a species complex with morphologically indistinguishable species, stands out among them as the most versatile and important insect vector of plant viruses [10,11]. *B. tabaci* transmits diverse plant pathogenic viruses with either DNA or RNA as the genetic material in a non-persistent, semi-persistent, or persistent mode (Table 1). However, only the DNA viruses belonging to the genus *Begomovirus* are known to be transmitted by whiteflies in a persistent-circulative manner [12,13]. Recently, certain viruses belonging to the non-whitefly transmitted genera *Polerovirus* and *Rhabdovirus* were shown to be anomalously transmitted by whiteflies, and the list continues to expand. However, the template minimal virus–whitefly interaction required for specific transmission of a virus by whitefly remains unknown. Here in this review, we summarize the known interactions of the whitefly with persistently transmitted begomoviruses and discuss new virus–whitefly relationships.

## 2. Transmission Route of Persistent Viruses

**Figure 1:**
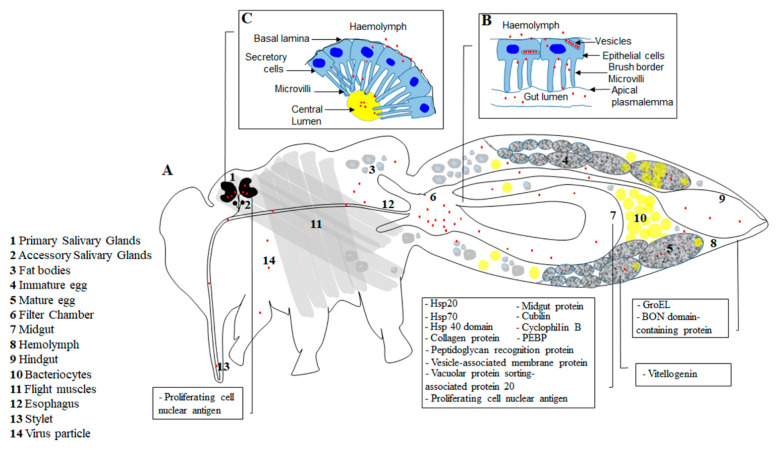
Whitefly internal anatomy (**A**) showing the major organs and proteins (in boxes) involved in circulative transmission of plant viruses by *B. tabaci*. The virus (red particles, number 14) is ingested with the phloem sap and is transported to the midgut (7) via the stylet (13) and esophagus (12). In the filter chamber area (6), the virus particles cross the midgut barrier (**B**) into the hemolymph (8) via the midgut plasmalemma and epithelial brush border. The virus circulates in the hemolymph and reaches the primary salivary glands (1), where it is internalized into the glands via the basal lamina and secretory cells to reach the central lumen that empties into the salivary glands duct (**C**). The virus then moves from the salivary glands duct into the salivary canal and is egested outside of the body while feeding. The accessory glands (2) have no role in the transmission. Some virus particles that do not reach the hemolymph are secreted outside of the body with the honeydew via the hindgut. The virus (TYLCV) has been shown to enter developing oocytes (4) and eggs (5), and could be transovarially transmitted to the next generation. Endosymbionts (10) play an important role in the transmission by secreting GroEL into the hemolymph (see text for details).

The transmission process of begomoviruses has been studied in detail and remains the main stay of our understanding of whitefly-mediated, persistent transmission of viruses. Although many other virus genera are transmitted by whiteflies in different transmission modes, little attention has been paid to the transmission mechanisms of other viruses. In this review, we thus use the known circulation process of the tomato yellow leaf curl virus (TYLCV) to describe the route of persistent, circulative viruses inside the whitefly. The transmission process of persistent viruses by whiteflies involves the following stages (Figure 1).

*Virus Ingestion*: Virus particles mixed with the phloem sap from infected plants are ingested by the piercing-sucking mouthparts to reach the cibarium. The cibarium of the whitefly attaches and accumulates the non-persistently and semi-persistently transmitted viruses, and sorts them from the circulative viruses that continue through the esophagus to reach the midgut lumen. Circulative viruses can be PCR-detected in the cibarium and midgut of the whitefly after a minimum of 5 and 40 min of feeding, respectively [26].

*Virus attachment in the midgut*: Recognition of the capsid protein [27] of only specific viruses by receptors is the first selection criteria that leads to virion adsorption on the apical plasmalemma of the anterior midgut or, specifically, the filter chamber [26,28,29]. The transmissibility of different begomoviruses by different whitefly species varies largely, and the receptors of the different begomoviruses remain mostly unidentified. Furthermore, the minimal criteria for recognition of a virus by the whitefly receptor remain elusive. Only two whitefly proteins, cubilin and amnionless, were recently found to form a receptor complex that binds TYLCV to the apical membrane [30]. Specific binding of the receptor proteins with the virus capsid proteins is critical for transmission. For example, saturation of midgut receptors by competing proteins impairs virus acquisition [27], whereas a lack of interaction between the midgut receptor and the capsids of a non-specific virus prevents virus entry into the cytoplasm of the midgut cells [29,31,32,33,34]. However, only small amounts of the persistent virus ingested is internalized from the lumen. Virus DNA remains detectable by PCR from the midgut for multiple days after acquisition access, indicating a storage mechanism [31]. Ingested virions are also egested along with the honeydew [35], especially the non-transmissible viruses which fail to attach to the midgut receptors [33,36,37].

*Virus entry and translocation across the basal membrane*: Successful recognition of specific begomoviruses triggers reorganization of the midgut actin filaments [38], resulting in the internalization of the virus into the midgut epithelial cells by clathrin-mediated endocytosis [29,39] into early endosomes [38]. Several whitefly proteins expressed in the midgut cells interact with the capsid protein of the virus to either facilitate or inhibit intracellular movement of the virus within the midgut cells (Table 2). Midguts were reported as a site of replication for TYLCV [40]. The minimal interactions within the midgut cells, or a consensus translocation process determining effective translocation across the epithelial cells, are yet unknown, as the specific role of these protein interactions for virus transmissibility remains to be characterized.

The infection of midguts with TYLCV alters the expression of genes that regulate cargo-receptor activity and ATP-binding cassette transporters, indicating their role in the translocation of the virus across the gut epithelial cells [59]. A recent study [38] disassociated the insect Golgi apparatus or late endosomes for any role in intracellular transport of TYLCV within the midgut cells, suggesting direct transport of the virus containing endosomes to the basal membrane by tubular vesicular structures induced by Snx12. However, successful translocation across the midgut into the hemolymph alone does not guarantee whitefly transmissibility of the virus, since the latter also relies on the rate of translocation [37,60]. The minimum time known for TYLCV to translocate across the midgut into the hemocoel is 90 min after acquisition access to the virus source (whitefly is caged with the source plant) [26].

Virus entry inside the midgut, in addition to altering whitefly gene expression for facilitating its spread, triggers an immune response. Genes encoding antimicrobial peptides such as knottins and defensins, humoral and cell surface receptors regulating cell defense, and intracellular signaling molecules involved in defense response are upregulated in the midguts following virus acquisition [59]. Activated synthesis of antimicrobial peptides inhibits TYLCV accumulation in *B. tabaci* [61]. Interestingly, infection with TYLCV simultaneously induces autophagy and apoptosis in *B. tabaci* midguts [52,59,62,63], albeit both pathways are found to be independent of each other. Genes regulating the autophagy–lysosome pathway such as autophagy related genes, cathepsins, and acid phosphatase are upregulated in the midguts following acquisition of TYLCV [59,62]. Similarly, viruses known to replicate inside *B. tabaci* were found to induce upregulation of apoptosis marker genes such as caspase-1/3 and downregulation of inhibitors of apoptosis such as bcl-2 [63]. Activation of autophagy-pathway-related genes inhibits TYLCV accumulation in *B. tabaci* [62], whereas induced apoptosis in whiteflies causes higher accumulation of the virus in the midgut, possibly by helping virions escape from degradation [63]. Phosphatidyl ethanolamine binding protein (PEBP) of the whitefly was recently reported as a regulatory protein coordinating the autophagy and apoptosis pathways to limit virus load from attaining levels that would affect host fitness [52]. The autophagy pathway of the whitefly is triggered with an accumulation of TYLCV up to a threshold level until the virus can replicate within the whitefly host [57,62]. Activation of autophagy genes with extended acquisition periods of whiteflies to TYLCV is a proposed reason as to why replication of TYLCV within the whitefly is only witnessed with shorter acquisition periods [40,57,62]. However, the defense response mechanisms of the whitefly against persistently transmitted viruses are yet to be understood clearly, and other immune pathways could also contribute to the decline in virus load over time within the whitefly.

*Retention and circulation in the hemocoel*: The hemolymph of the whitefly remains the most understudied barrier, although it is thought to be a hostile environment that challenges the stability of virions. No interactions of the virus with any hemolymph proteins, hemocytes other than with the whitefly vitellogenin protein [44], or proteins secreted by the endosymbionts [31,53,54,56] are known (Table 2). Interaction with vitellogenin is suggested to be crucial for the transovarial transmission of some begomoviruses onto oocytes [44]. Direct interactions of viruses with endosymbionts scattered in the hemolymph, or indirect transmission with secreted proteins of endosymbionts localized within the bacteriosomes, have been implied to affect virus transmission in multiple studies [53,56,64,65,66]. The interaction of the GroEL protein of the bacterial symbionts with virus capsids is highlighted in its hypothesized role in the protection of virions while they circulate in the hemolymph [31,53,54] until they reach the periphery of the primary salivary glands. The exact role and mechanisms through which bacterial endosymbionts influence virus transmission is yet to be elucidated. Frequent positioning of the whitefly midgut in the thorax to accommodate eggs may reduce the required circulation distance for virions to reach the salivary glands [67,68] and aid transmission. Viruses were previously detected within fat cells [69] circulating in whitefly hemolymph; however, whether they have a role in transmission is yet unknown.

*Translocation across the membranes of the salivary gland*: After circulating in the hemocoel, the virions cross the basal lamina of the primary salivary glands (PSG) of the whitefly [34,67,70]. The minimum time required for the detection of TYLCV in the salivary glands is 7 h from acquisition [26]. The PSG were recently reported as the one organ besides the midgut that supports replication of TYLCV by interacting with the whitefly proliferating cell nuclear antigen protein (Table 2) and recruiting the host DNA replication machinery [57]. However, entry of virions inside the PSG does not guarantee its transmission by whiteflies [71,72]. The PSG are kidney-shaped organs differentiated into a central region and two endcaps directed apically toward the central region [67]. The central region of the salivary glands consists of secretory cells of different sizes surrounding a central lumen (or internal duct) lined with microvilli that empties into an external duct joining the accessory salivary gland [70]. Receptor-mediated recognition of viruses leading to virus accumulation in the internal ducts of the PSG before being secreted along with saliva is a crucial step for transmission [72]. However, the role of virus-specific receptors or other factors affecting the stability of viruses inside the PSG is yet to be unraveled. Virions mixed in saliva are secreted through the salivary ducts to the salivary canal in the stylet and released inside the plant phloem cells while salivating. The minimum time required for inoculation of TYLCV to plants by whiteflies is 8 h after acquisition [26].

## 3. Viral Structural Proteins: The Key Determining Whitefly Transmissibility

Until recently, begomoviruses were not known to replicate within their insect vectors, and no virus-encoded helper protein is known to be essential for transmission. The role of the capsid protein (CP) of begomoviruses has thus been the most studied in relation to whitefly transmission, and evidence of its criticality in persistent whitefly transmission is provided in multiple studies involving diverse virus–whitefly combinations [73,74,75,76]. Although the template minimal sequence of the CP determining the specific transmissibility by different whitefly species remains unknown, the region of the CP of begomoviruses between the conserved amino acid domains GCEGPCKVQS and LYMACTHASN [37,72], or more specifically between amino acids 129 and 152 [71], is critical for whitefly transmission. Single point mutations within this region of the CP were reported to render the mutants non-transmissible by whiteflies [71,76,77], or result in altered transmission efficiency by the different whitefly species [78]. Similarly, swapping of fragments between these conserved domains of CP between a transmissible and non-transmissible virus resulted in transmission of the non-transmissible virus, and vice versa [72]. The loss of whitefly transmissibility with mutations within the critical region has been attributed to altered capsid structure, leading to a loss in the ability to cross the midgut or salivary gland barriers and instability in the hemolymph [71,72,76,78].

## 4. Regulation of Virus Transmission

Multiple pieces of evidence for replication of TYLCV inside whiteflies [40,62] imply that TYLCV virions disassemble and enter the whitefly host cell nuclei. Evidence of transcription of TYLCV within whiteflies was previously reported [79], but the recent association of the replication-associated protein (RepA) of TYLCV with the whitefly DNA replication machinery inside whitefly salivary glands [57] implicates active translation of TYLCV, as the RepA proteins must be encoded within the whiteflies. The transcription of the viral DNA inside plant nuclei is regulated by transcription factors of host plants that bind to virus DNA promoter sequences at the intergenic region of the virus genome [80,81]. Similarly, transcription of TYLCV inside the whitefly is known to be regulated by at least three different whitefly-encoded transcription factors [82]. On the other hand, transcription in virus-infected plants is also altered by the presence of viral proteins. The CP protein of a begomovirus shuttles viral DNA in and out of the host nuclei [83,84], often localized inside the plant and insect cell nuclei [83,84,85], and exhibits DNA binding activity [86] through which it regulates transcription of its host plant. For example, the interaction between viral proteins and host plant transcription factors often suppresses the host plant defenses and results in positive repercussions for vector fitness [87,88]. Similarly, the gene expression of the whitefly is altered following virus acquisition. Perturbation of the whitefly cell cycle and metabolic pathways or activation of whitefly immune response genes following virus acquisition was reported in multiple studies [59,69,89,90,91], indicating intricate interactions between the virus and whitefly. Whether and how the whitefly gene expression is regulated by the interaction of viral proteins with whitefly transcription factors and activators is yet unknown. Plants employ epigenetic strategies such as viral chromatin methylation as a defense mechanism against viruses [92,93], whereas virus proteins are known to inhibit global methylation to counter the plant defenses [94]. The molecular basis of the regulation of viruses within the whitefly remain unknown; however, such interactions are observed in other insect–pathogen systems. For example, the transcription of multiple mosquito genes related to the transmission of *Plasmodium*, the malarial parasite, is regulated by histone modifications in the chromatin of the mosquito [95], and *Plasmodium* infection induces histone modifications in genes regulating vector competence [96]. Enzymes catalyzing DNA methylation in invertebrates, such as DNA methyltransferases 1 and 3, are characterized from *B. tabaci* and are implied to regulate temperature tolerance [97,98]. Future characterization of whitefly transcription regulatory elements that interact with the viral proteins could be crucial to understanding the replication of only certain viruses inside the whiteflies, and anti-viral responses.

## 5. Evolution of New Relationships and Associated Risk

Poleroviruses (Luteoviridae) comprising at least 31 species were erstwhile known to be strictly transmitted by aphids in a persistent non-propagative manner [4,23,99]. However, at least two new recombinant poleroviruses, pepper whitefly-borne vein yellows virus (PeWBVYV) and cucurbit whitefly-borne yellows virus (CWBYV), have been reported to be transmissible exclusively by the MEAM1 species of *B. tabaci* [23,24]. The transmission route and tissue specificity of poleroviruses inside the aphids are quite similar to those of begomoviruses in whiteflies [99]. PeWBVYV also follows a circulative pathway inside *B. tabaci,* and translocates across the midgut onto the hemolymph; however, it circulates inside the whitefly for a longer duration (≥120 h) before transmission [58]. Whether the circulative transmission of poleroviruses inside the whitefly involves the same interactions as that of begomoviruses remains to be characterized. The genomes of the recombinant poleroviruses transmitted by *B. tabaci* are highly identical to those of other poleroviruses transmitted by aphids, but the factors that determine the specific transmission of poleroviruses by aphids or whiteflies remain unknown. Moreover, no common pattern linked to the whitefly transmission can be identified by comparison of the virus-encoded proteins of PeWBVYV and CWBYV [24]. The two structural proteins of the poleroviruses, the major capsid protein (CP) and the read-through minor capsid protein (RTD), are important for recognition by aphid receptors [100] and transmissibility [101], respectively. The CP and RTD of PeWBVYV and CWBVYV largely differ from their close aphid-transmitted relatives, suggesting a role in altered whitefly transmission. Additionally, the cross-transmission of the aphid transmitted polerovirus by whiteflies from plants co-infected with the aphid- and whitefly-transmitted viruses also indicates the involvement of the capsid proteins as the factor determining its transmission [102]. Complement component 1Q subcomponent-binding protein (C1QBP), a hexameric glycoprotein of whitefly and aphid, was found to interact with the major capsid protein of PeWBVYV [58]. Moreover, C1QBP protein of the aphids interact with the capsid proteins of aphid transmitted poleroviruses [58,103]. Although the exact role remains unknown, yet the interaction of whitefly/aphid C1QBP with polerovirus capsid proteins further indicates the usage of common machinery by persistent non-propagative viruses for transmission by their insect vectors.

The emergence of poleroviruses transmitted by *B. tabaci* can have severe ramifications on global crop production. For example, the spread of PeWBVYV by whiteflies in Israel has forced the growers to completely abandon open-air cultivation and re-equip net-houses with whitefly-proof mesh. The lack of resistant cultivars against PeWBVYV has led to the heavy usage of insecticides from the beginning of the season to control whitefly, resulting in diminished economic returns. PeWBVYV was absent in the Arava valley region, the main pepper-growing area of Israel until the 2019 season, but has been recently detected in sporadic places (unpublished) and continues to threaten pepper cultivation in Israel. However, the evolutionary pressure necessitating the development of this new *B. tabaci*–polerovirus relationship is yet to be understood. A recent study suggests that the transmission of highly similar poleroviruses by different insect vectors is a strategy of competing viruses to escape competitive exclusion [102]. The back-to-back identification of the two whitefly-transmitted poleroviruses in two very different crops (PeWBVYV in pepper and CWBVYV in melons) from Israel and Brazil, respectively, suggests that the emergence of *B. tabaci* transmitted poleroviruses is independent of the crop and geographical location. Crops belonging to diverse families such as cucurbits, beet, solanaceous crops (pepper, eggplant, tobacco, and potato), sweet potato, and cotton support high whitefly populations and are known hosts for multiple poleroviruses, putting them at high risk for the potential emergence of *B. tabaci* transmitted poleroviruses. Moreover, the worldwide spread of the polyphagous Middle-East Asia Minor 1 (MEAM1) species of *B. tabaci* intensifies the threat, and warrants monitoring for the association of whitefly incidence and viral disease symptoms. Lack of awareness of the whitefly as a possible vector can lead to rapid spread of the virus disease, leading to the introduction of the virus into new areas.

The recent report of the transmission of the legume-infecting bean-associated cytorhabdovirus (BaCV) belonging to Rhabdoviridae in Brazil by *B. tabaci* [25] is another new addition to the diversity of plant viruses transmissible by whiteflies. Rhabdoviruses, apart from the fungi-transmitted varicosaviruses, were only known to be transmitted by leaf hoppers, plant hoppers, aphids, or mites in a persistent propagative mode [104]. Furthermore, the genome sequence of BaCV is similar to that of viruses previously identified from *B. tabaci* in India, bean crops in Tanzania, and multiple crops in China, suggesting widespread existence of *B. tabaci* transmitted rhabdoviruses [25]. Future confirmation of whitefly transmission of such viruses genetically similar to BaCV would be the first step before assessing the potential risks associated with whitefly transmission of rhabdoviruses. Moreover, future answers about the rhabdovirus propagation or its circulative route within the whitefly would enrich the understanding of the diverse mechanisms used by the whitefly to serve as vectors of plant viruses.

## 6. Is It MEAM1 Breaking Barriers?

The MEAM1 species of *B. tabaci* is highly polyphagous, prevalent in all the continents (except Antarctica), has moderate to high levels of resistance to insecticides [105], and is especially noted for efficient virus transmission. The whitefly transmission of poleroviruses (PeWBVYV and CWBVYV) and rhabdovirus (BaCV) was reported using the MEAM1 species of *B. tabaci*. Interestingly, the Mediterranean (MED) species of *B. tabaci*, which is prevalent in Israel in addition to MEAM1, was reported as a non-transmitter of PeWBVYV [58]. PeWBVYV was translocated across the midgut into the hemolymph of both MEAM1 and MED, but the virus titers were significantly lower in the hemolymph of the latter. However, the specific factors hindering the transmission of PeWBVYV by MED still remain an enigma to be solved. Comparative transmission of CWBVYV and BaCV with different species of *B. tabaci* would shed more light on whether MEAM1 has a special contribution toward the emergence of *B. tabaci* as a vector of new viruses.

## 7. Concluding Remarks

The specific transmission of circulative viruses by specific whitefly species involves intricate interactions between the virus and the whitefly. The minimal requirement of interactions between the virus and the whitefly receptors to ensure whitefly transmissibility is yet to be deciphered. Several whitefly proteins identified to interact with the virus are implied to serve a role in transmission; yet, comparative studies with the identified protein–protein interactions in different whitefly–virus systems are required to understand the minimal requisite interactions to effectuate virus transmission. Moreover, the absence of a *B. tabaci* cell-line has been a major stumbling block for the conclusive identification of receptors or mechanisms of intracellular trafficking of the virus inside midgut or salivary glands. Understanding the regulation of gene expression of *B. tabaci* by acquired viruses can be key to deciphering the genes governing antiviral immunity. New relationships have evolved between whiteflies and viruses belonging to families conventionally transmitted by other insects, resulting in the increased threat of whiteflies as a quarantine pest. Prompt risk-assessment of whiteflies as non-conventional transmitters of viruses is required to mitigate the spread and evolution of emerging whitefly-transmitted virus diseases.

## Figures and Tables

**Table 1:** List of the genera of viruses transmitted by whitefly.

Genus	Family	Genetic Material	Mode of Transmission	No. of Species Transmitted by Whitefly	References
*Begomovirus*	Geminiviridae	ssDNA	Circulative, non-propagative	424 ^a^	
*Crinivirus*	Closteroviridae	(+)ve ssRNA	Semi-persistent	14 ^a^	
*Ipomovirus*	Potyviridae	(+)ve ssRNA	Semi-persistent	7	[14,15]
*Torradovirus*	Secoviridae	(+)ve ssRNA	Semi-persistent	5	[16,17,18]
*Carlavirus*	Betaflexiviridae	(+)ve ssRNA	Non-persistent	4	[19,20,21,22]
*Polerovirus*	Luteoviridae	(+)ve ssRNA	Circulative, non-propagative	2	[23,24]
*Cytorhabdovirus*	Rhabdoviridae	(–)ve ssRNA	Unknown	1	[25]
Total viruses		457			

^a^ Number of species listed in the ICTV 2019 release.

**Table 2:** Protein–protein interactions between the whitefly and circulative viruses.

Virus Protein	Whitefly Protein	Localized Organelle of Interaction	Suggested Site of Interaction	Suggested Role	References
CP	Heat shock proteins Hsp20, Hsp70	Midgut		Inhibits virus inside whitefly	[41,42]
CP	Cyclophilin B	Midgut, salivary gland, ovary	Midgut, ovary, salivary gland	Aids virus to suppress whitefly immune response	[43]
CP	Cubilin	Midgut	Midgut	Receptor for entry into midgut cells	[30]
CP	Vitellogenin	Ovary	Hemolymph, Ovary	Viral entry into ovary cells for transovarial transmission	[44]
CP	Peptidoglycan recognition protein	Midgut		Whitefly immunity against virus	[45]
CP	Midgut protein (transcription activator MBF2 protein domain)	Midgut	Midgut	Aids translocation of virus across midgut	[46]
CP	Vesicle-associated membrane protein-associated protein B	Midgut	Midgut	Inhibits virus translocation across midgut	[47]
CP	Vacuolar protein sorting-associated protein 20		Midgut	Aids virus translocation across midgut	[48]
CP	Thioredoxin like protein				[49]
	Collagen protein	Midgut	Midgut	Aids adhesion and entry to the midgut epithelial cells	[50]
	Tumerous imaginal disc protein (Hsp 40 domain)		Midgut	Inhibits virus inside whitefly	[51]
CP	Phosphatidylethonolamine-binding protein (PEBP)			Immune homeostasis by regulation of autophagy and apoptosis	[52]
CP	GroEL * (Hamiltonella, Arsenophonus)		Hemolymph	Protects virions within hemolymph	[53,54,55]
CP	BON domain containing protein (Rickettsia) *		Hemolymph	Protects virions within hemolymph	[56]
Rep	Proliferating cell nuclear antigen		Salivary gland, midgut	Aids viral replication	[57]
CP (polerovirus)	C1QBP				[58]

* *B. tabaci* endosymbiont proteins.
